# Machine learning model optimization for compressional sonic log prediction using well logs in Shahd SE field, Western Desert, Egypt

**DOI:** 10.1038/s41598-025-97938-9

**Published:** 2025-04-29

**Authors:** Khaled Saleh, Walid M. Mabrouk, Ahmed Metwally

**Affiliations:** 1 Geophysics Department, Faculty of Science, Cairo University, Giza, Egypt; 2 PetroShahd Company, Zahraa Maadi, Cairo, Egypt

**Keywords:** Compressional sonic, Machine learning, Sonic prediction, Logs prediction, Geophysics, Geology

## Abstract

Compressional sonic logs is one of the important logs for subsurface characterization, reservoir evaluation, and wellbore stability analysis. However, acquiring these logs is often challenging due to logistical constraints. This study explores the application of machine learning (ML) techniques to predict compressional sonic logs using conventional well logs from five wells. The methodology involves data preprocessing, feature selection, and training various regression models, including Random Forest, CatBoost, XGBoost, K-Nearest Neighbors (KNN), Support Vector Machines (SVM), and Deep Neural Networks (DNN). Model performance is optimized through hyperparameter tuning and evaluated using correlation coefficients and root mean square error (RMSE) metrics. Results indicate that ensemble models (Random Forest, CatBoost, XGBoost) achieve the highest accuracy, with correlation coefficients ranging from 89 to 89.6% and RMSE between 5.85 and 6.03. Additionally, feature engineering and data cleaning significantly improve model performance, while input scaling is essential for SVM, KNN, and DNN models. Incorporating blind well testing further enhances reliability. This study presents a robust ML-based workflow for predicting compressional sonic logs, offering a cost-effective solution for reservoir management and geomechanical analysis.

## Introduction

Compressional sonic logs is considered to be one of the most important logs in subsurface characterization across geology, geophysics, petrophysics, and petroleum engineering. They measure the travel time of compressional (P-wave) and shear (S-wave) acoustic waves through rock formations, providing valuable data on lithology, porosity, and mechanical properties. In geophysics, sonic logs are used for seismic calibration, enabling the generation of synthetic seismograms for improved seismic^1^. In petrophysics, sonic logs assist in porosity estimation using the Wyllie time-average equation and help evaluate rock mechanical properties such as Young’s modulus and Poisson’s ratio^2^. In petroleum engineering, compressional sonic logs aid in wellbore stability analysis, fracture identification, and real-time drilling optimization^3^. Their widespread application makes them indispensable in hydrocarbon exploration and reservoir characterization.

**Fig. 1:**
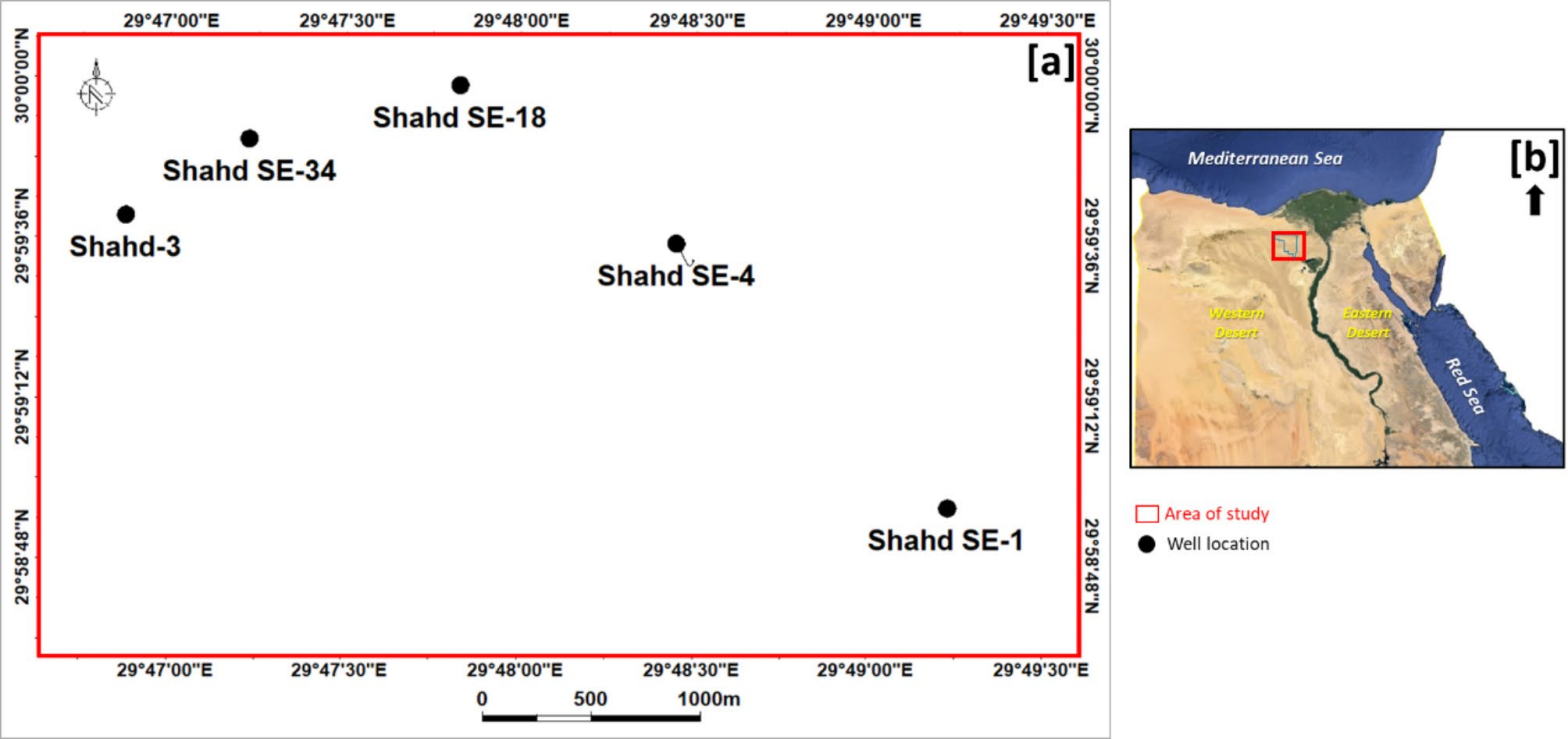
Base map showing (**a**) the distribution of well locations and (**b**) the study area within the Western Desert of Egypt.

The Shahd SE field is located in the north eastern part of the Western Desert of Egypt (Fig. 1). This field is considered to be primary producing field in PetroShahd Company. Despite numerous wells drilled and high-resolution seismic data acquired, miss match between prognosed formation tops from seismic cubes and actual drilling results still persist. With the sonic log, geophysicists can create synthetic seismograms, adjust seismic horizons, and fine-tune velocity models which will be helpful in the interpretation of subsurface features.

Moreover, hydraulic fracturing is deemed necessary for the Upper Bahariya Formation, the secondary reservoirs within the field. Compressional sonic data holds significant utility for synthetic seismograms, velocity modeling^4^, shear sonic prediction^5^ and guiding hydraulic fracturing design^6^.

The estimation of compressional wave velocity (Vp) is achieved through direct logging measurements, empirical models, and advanced computational techniques. Wireline sonic logging and logging-while-drilling (LWD) tools provide direct measurements of P-wave travel time. Despite the high-resolution data provided by direct sonic log measurements for formation evaluation, their acquisition involves significant costs and additional rig time. Moreover, prolonged exposure of shaly formations to open-hole conditions during logging operations can compromise wellbore stability, increasing the risk of borehole collapse and formation damage^7^. Empirical models, such as the Wyllie time-average equation and Raymer-Hunt-Gardner model, estimate Vp based on porosity and lithology-dependent factors^8,9^. Additionally, machine learning techniques, including artificial neural networks (ANNs) and deep learning models, enable the reconstruction of missing sonic logs by leveraging relationships between petrophysical well logs^10^. Seismic-derived methods, such as velocity inversion and rock physics templates, integrate well-log data with seismic attributes to estimate Vp in regions lacking direct measurements^11,12^. Machine learning offers a solution by accurately predicting compressional velocities using vast datasets^5,13^. This study aims to develop a workflow for generating compressional sonic logs using various machine learning algorithms and optimizing the most effective model for implementation.

This paper explores the integration of machine learning into compressional sonic log prediction to overcome data collection challenges and provide valuable insights for subsurface characterization and reservoir management. The objective is to produce high-resolution, high-accuracy, and spike-free compressional sonic logs by leveraging available legacy data, eliminating the need for additional logging tools, and avoiding extra operational costs.

### Methodology

The methodology involves data collection of the available logs for the five wells in the study in the zone of interest from A/R “F” member to top of Kharita formation. Cleaning and preprocessing, including imputation of the missing values and handling inconsistencies. Facies interpretation is performed using existing logs and mud log data, incorporating an additional feature that has the potential to enhance model performance.

The next step is the features selection in which the relevant features, such as gamma ray, resistivity, density, neutron, and photoelectric factor logs. Additional features engineering processes such as transformation and normalization is done if necessary. The selected features are divided among four wells for use in training and testing sets, while the fifth well is designated as a blind test well.

**Fig. 2:**
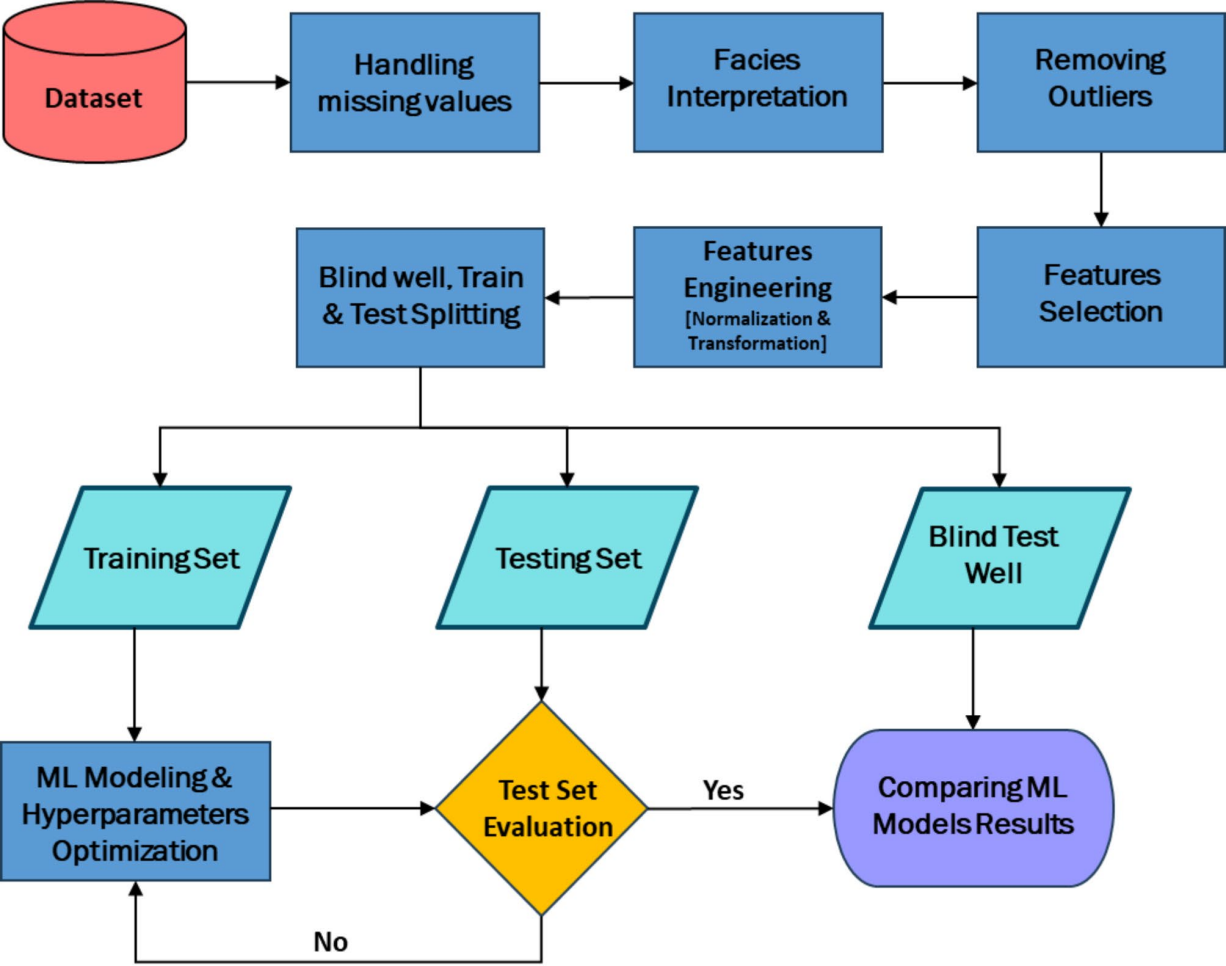
Machine Learning Workflow for compressional sonic log prediction: From data preprocessing to model training, evaluation, and comparison.

Various types of machine learning regression algorithms, including Random Forest, CatBoost, XGBoost, nearest neighbors (KNN), Support Vector Machines (SVM), and Deep Neural Networks (DNN), are explored and experimented. Each model is trained using the same training set and hyperparameters are optimized to maximize predictive performance. Finally, the model performance is evaluated using appropriate metrics using testing set and blind well (Fig. 2).

### Data collection and preprocessing

**Fig. 3:**
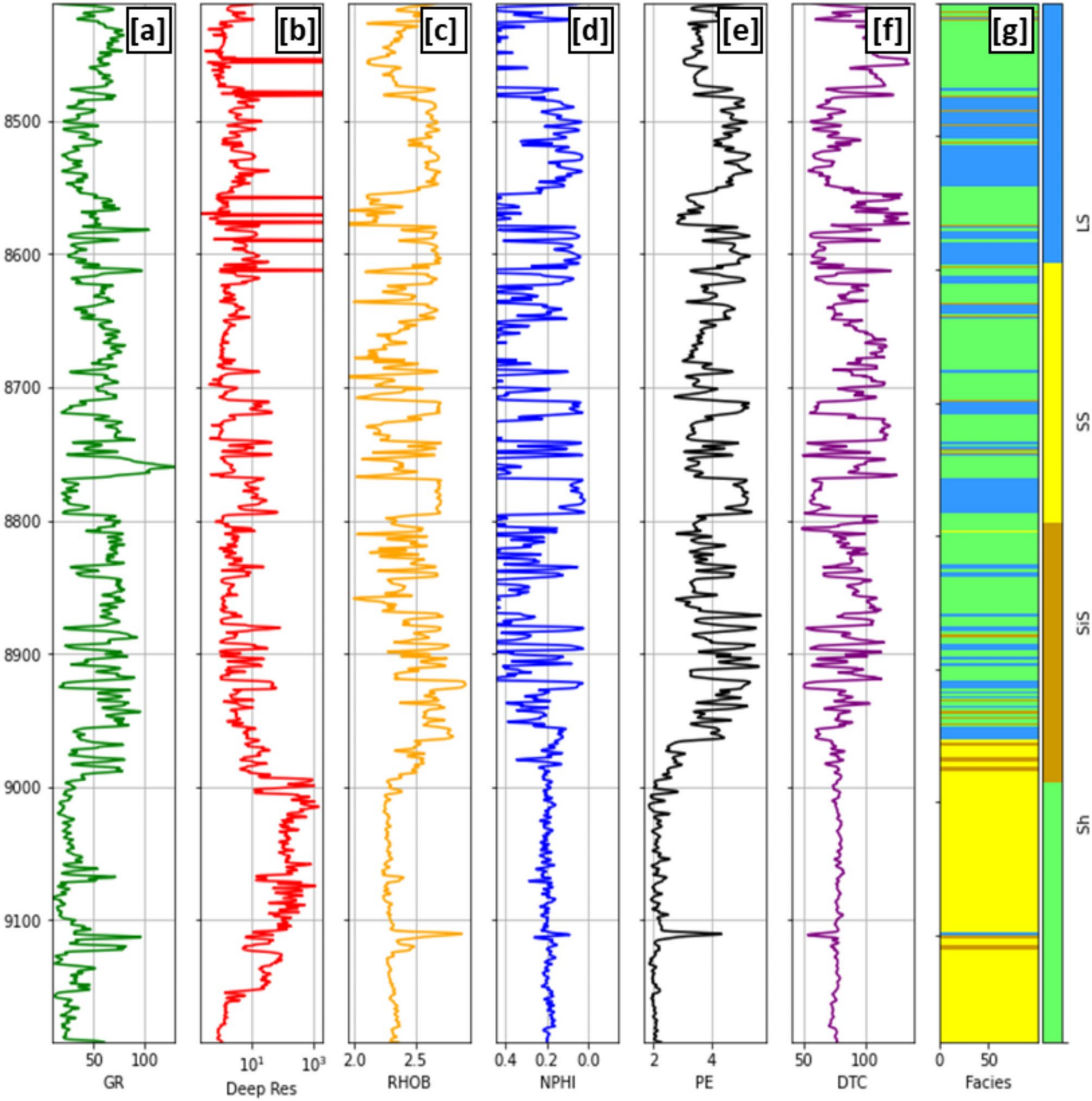
Shahd SE-08 well (**a**) Gamma ray (GR), (**b**) Deep resistivity (Deep_Res), (**c**) Density (RHOB), (**d**) Neutron (NPHI), (**e**) Photoelectric Factor (PE), (**f**) Compressional sonic [DTC] and (**g**) Facies interpretation log (Sh: Shale, SS: Sandstone, SiS: Siltstone, LS: Limestone) versus depth.

This study utilized data from five wells. The available E-logs for these wells included Gamma ray (GR), Density (RHOB), Deep Resistivity (Deep_Res), Neutron (NPHI), Photoelectric Factor (PE), and Compressional Sonic (DTC) logs (Fig. 3). The data from these wells were collected over a consistent interval, ranging from the A/R “F” member to the top of the Kharita Formation, thereby covering the primary zone of interest.

Machine learning models require a complete dataset, meaning missing values in input features or the target variable must be addressed before training. To retain as much data as possible, missing values were imputed using the average of neighboring data points rather than removing entire rows. This method is justified by the high sampling resolution of 0.5 ft, where geological property variations are minimal over such short intervals. Furthermore, the occurrence of continuous missing values was limited to no more than two consecutive points, ensuring that interpolation effects remained minimal. The overall proportion of missing values was low, ranging between 0.5% and 1.3%, reducing the potential impact of imputation on model performance. This approach balances data completeness with geological consistency, ensuring that valuable training information is preserved while minimizing distortions in the dataset.

A new feature has been added to the dataset which can be obtained from the original logged data. Litho-facies log have been interpreted based on the conventional E-logs and the samples description obtained from the mud log (Fig. 3g). Four lithologies have been identified throughout the interested zone which are shale, siltstone, sandstone and limestone.

Outliers can notably affect the of ML models performance by biasing the estimation of model parameters, which can result in inaccurate predictions. Furthermore, outliers can diminish the robustness of machine learning models, rendering them less dependable in practice where data points may contain noise or anomalies^14^.

Table 1 presents the statistical analysis of the logs, detailing the range of data points for the raw logs. Outliers can be detected through geological knowledge of the study area. For example, the lithology in the region of interest includes sandstone, shale, siltstone, and limestone. The typical density ranges for these lithologies are as follows: sandstone (2.0–2.6 g/cm^3^), shale (2.0–2.7 g/cm^3^), and limestone (2.5–2.8 g/cm^3^)^15^. Based on these ranges, density values below or above these ranges should be scrutinized. In contrast, Table 1 shows minimum and maximum observed values for density 1.389 and 3.14 respectively. Similarly, the neutron log in the study area shows a maximum value of 1.045, which exceeds the usual maximum range for neutron porosity values (0.6–0.7) seen in most formations. Neutron porosity values greater than typical formation porosities are often flagged for further inspection, as these may indicate measurement errors or special circumstances such as gas zones or tool malfunctions.

**Table 1:** Descriptive statistics of the original input features and target variable.

	GR	Deep_Res	NPHI	RHOB	PE	DTC
Mean	54.08803	351.75	0.224281	2.467634	3.351667	79.01904
Std	24.98756	5585.268	0.128468	0.16912	1.093514	15.12602
Minimum	8.299026	0.213875	0.0136	1.3898	1.2875	38.0698
25%	32.81696	1.00755	0.149913	2.3451	2.346162	70.4854
50%	52.5881	2.358125	0.18955	2.469425	3.33595	75.4263
75%	71.87499	7.2034	0.286887	2.600625	4.266325	86.13321
Maximum	218.129	100,000	1.0454	3.1401	8.321	137.8296

**Fig. 4:**
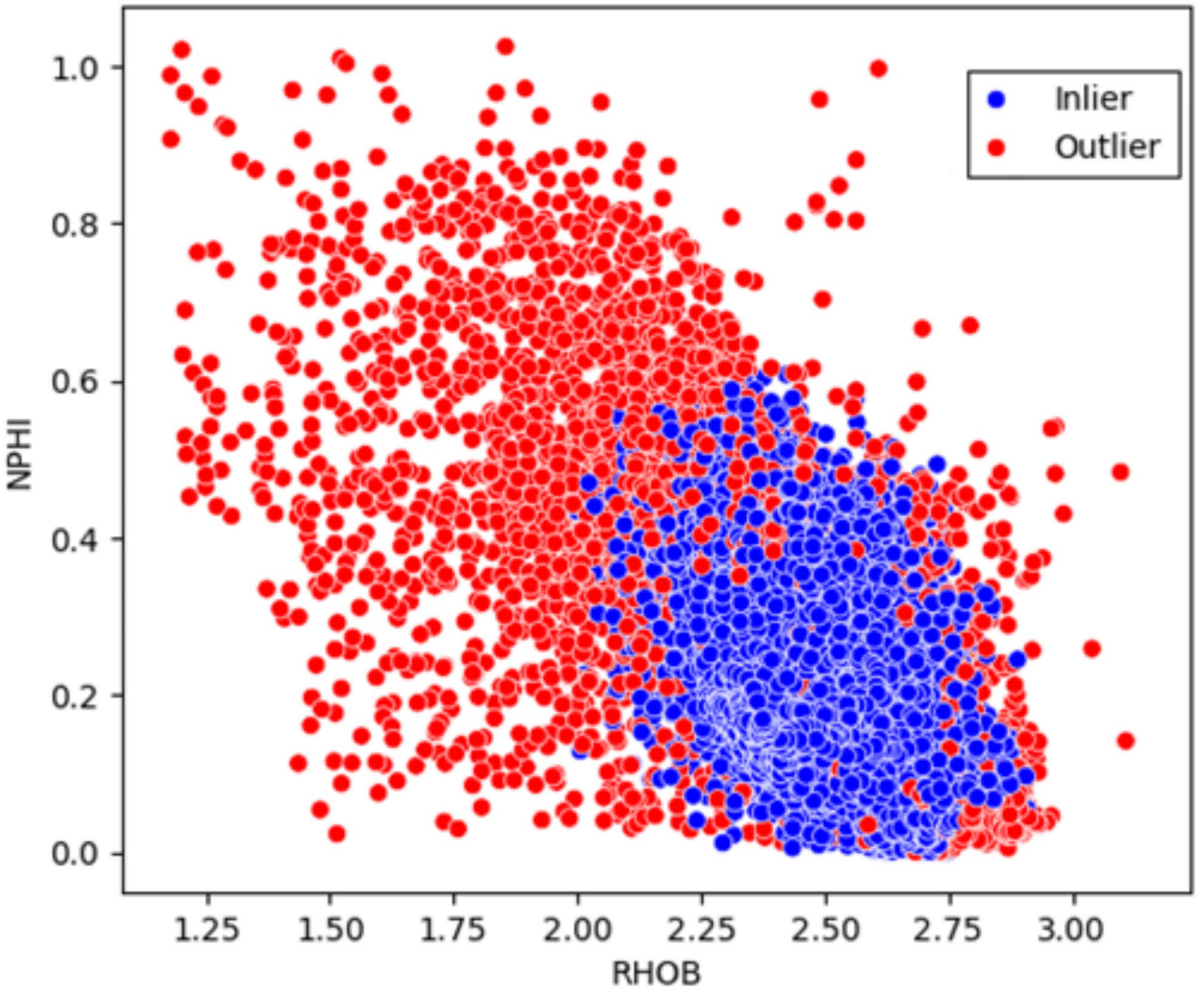
Crossplot of Density (RHOB) versus Neutron Porosity (NPHI), highlighting the inliers (blue) and the outliers (red).

To address and eliminate outliers and inaccurate data points, Isolation Forest algorithm was applied to the logs for all wells. The Isolation Forest algorithm, introduced by Liu et al.^16^, is an efficient and effective method for anomaly detection that isolates outliers using a tree-based approach^16^. Unlike traditional distance-based methods, Isolation Forest works by randomly selecting features and splitting data points recursively, leading to shorter paths for anomalies since they are more susceptible to isolation. This makes the algorithm computationally efficient, with a linear time complexity in relation to the number of samples^17^. Additionally, it performs well on high-dimensional data without requiring extensive preprocessing or dimensionality reduction. Figure 4 presents the crossplot of the density log versus the neutron log, illustrating the effect of applying the Isolation Forest algorithm for automatic outlier detection and removal. The algorithm successfully identified and eliminated 10% of the data as outliers, resulting in a refined dataset where the log values fall within the expected normal range, as detailed in Table 2. The most significant impact was observed in the upper range of the logs, with the maximum neutron log value decreasing from 1.04 to 0.63 and the maximum density log value reducing from 3.14 to 2.9. Additionally, notable changes were observed in the mean and standard deviation of both logs, which are critical for subsequent normalization steps in feature engineering.

**Table 2:** Descriptive statistics of input features and target variable.

	GR	Deep_Res	NPHI	RHOB	PE	DTC
Mean	47.16294	5.317307	0.186604	2.506769	3.707708	74.40674
Std	24.0113	7.507866	0.11432	0.136942	1.120721	14.15735
Minimum	5.6023	0.2616	0.0143	2.0003	1.345	36.13
25%	27.15457	1.132175	0.1034	2.3866	2.72	64.364
50%	41.35675	2.39805	0.1676	2.5144	3.80685	72.5695
75%	66.69	6.181875	0.243475	2.629098	4.771275	81.01158
Maximum	167.1226	83.4469	0.6303	2.9082	7.1282	126.3898

### Feature selection and engineering

Selection of features is an important aspect in machine learning model development, as it directly impacts the model’s performance, interpretability, and computational efficiency. The selected features should be relevant to the prediction task at hand. Features should have a meaningful relationship with the target variable and contribute useful information for making predictions^18^. The factors such as lithology, grain density, porosity, permeability, shale volume, grain size distribution, mineral composition and water saturation play a significantly role in affecting the rock properties^19–21^.

Statistical tests such as correlation analysis or mutual information can help assess the relevance of features^22^. The type of correlation calculated here is the Pearson correlation coefficient, which measures the linear relationship between two variables. The Pearson correlation coefficient, denoted as r, is calculated using the following equation:

1 $$r = \frac{{\mathop \sum \nolimits_{i = 1}^{n} \left( {x_{i} - \overline{x}} \right)\left( {y_{i} - \overline{y}} \right)}}{{\sqrt {\mathop \sum \nolimits_{i = 1}^{n} \left( {x_{i} - \overline{x}} \right)^{2} \mathop \sum \nolimits_{i = 1}^{n} \left( {y_{i} - \overline{y}} \right)^{2} } }}$$

where ${ }x_{i}$ and $y_{i}$ are the values of the two variables being compared, $\overline{x}$ and $\overline{y}$ are the means of the respective variables and $n$ is the number of paired scores.

A Pearson correlation coefficient close to + 1 indicates a strong positive linear relationship, whereas a value near − 1 suggests a strong negative linear relationship, and a value near 0 implies no linear correlation between the variables^23^

**Fig. 5:**
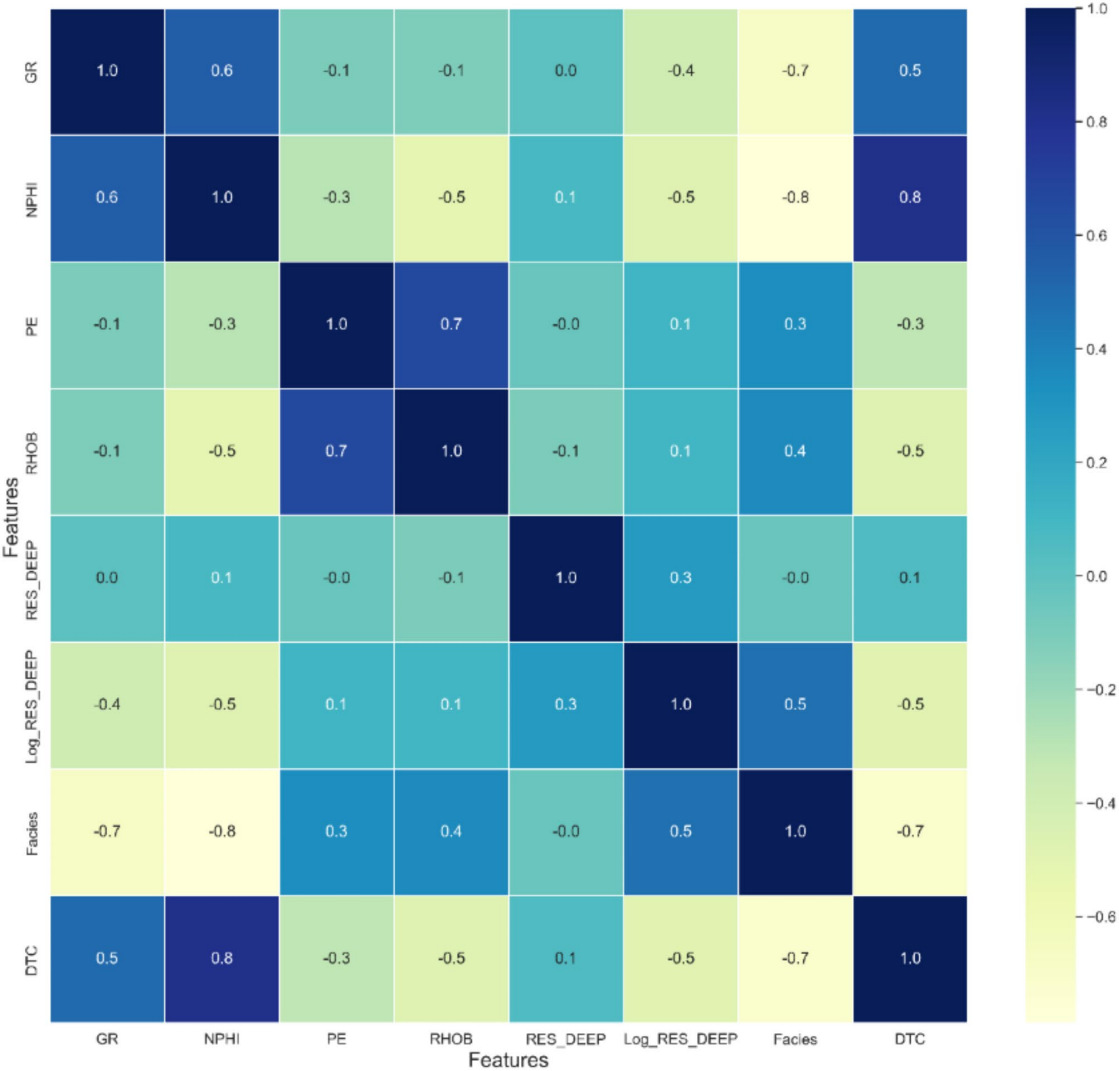
Correlation matrix for the input features and compressional sonic.

Figure 5 demonstrates that most features exhibit a correlation with the compressional sonic log, with the exception of the deep resistivity log. The neutron and gamma ray logs show strong positive correlations of 0.8 and 0.5, respectively, while the density and photoelectric factor logs exhibit moderate negative correlations of − 0.5 and − 0.3. Although fluid properties and saturation generally contribute to an increase in compressional-wave velocity^24,25^, in this study, the deep resistivity log shows a weak correlation of 0.1 with the compressional sonic log. However, this correlation improves to − 0.5 when using the logarithm of the deep resistivity log (Fig. 5). Consequently, the logarithm of the deep resistivity log is selected as an input feature instead of the original values, as this transformation helps reduce the dynamic range of the chargeability curve^26^.

**Fig. 6:**
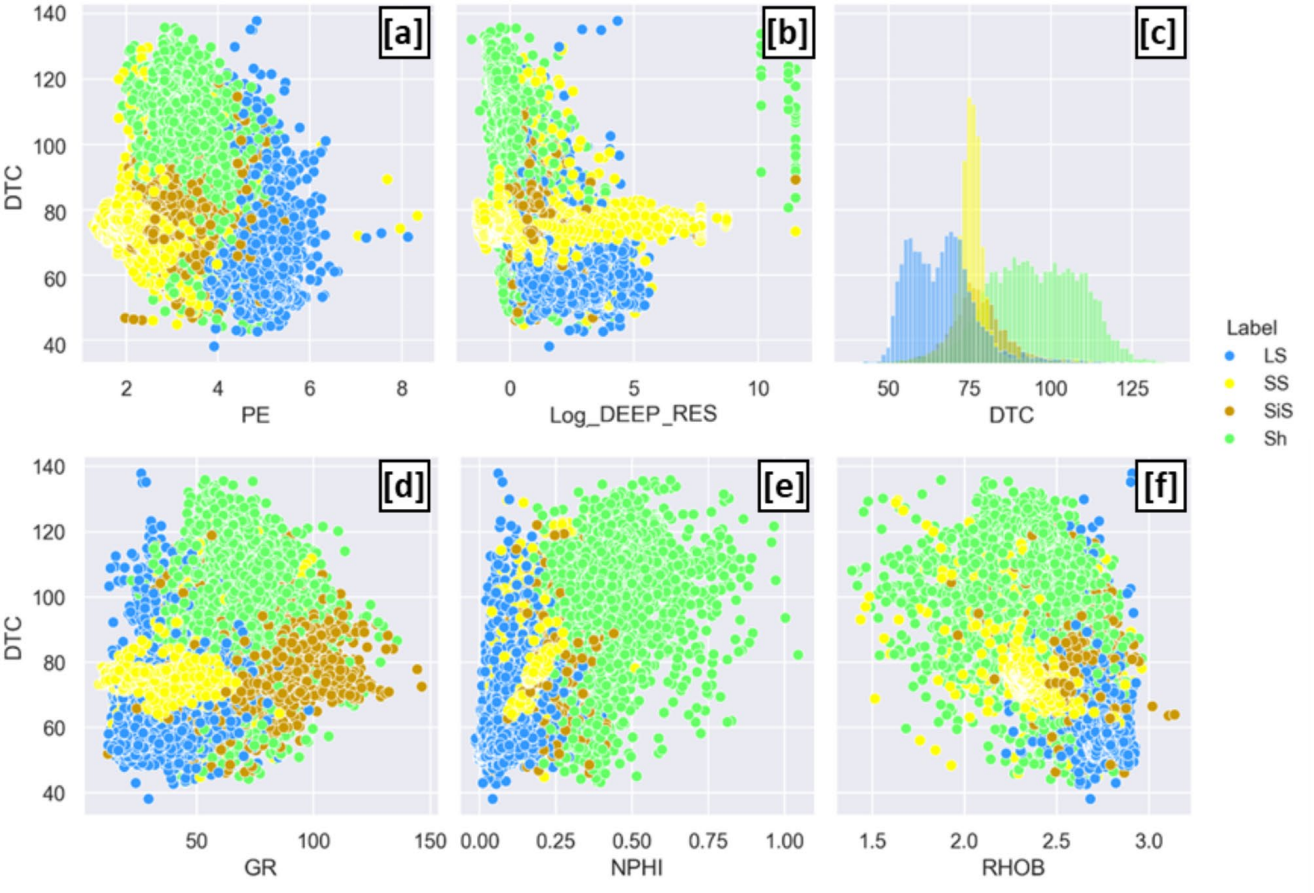
Cross plot of measured compressional sonic versus (**a**) Photoelectric Factor (PE), (**b**) Logarithm of Deep resistivity (Log_Deep_Res), (**c**) Compressional sonic [DTC] (Histogram), (**d**) Gamma ray (GR), (**e**) Neutron (NPHI), (**f**) Density (RHOB) labeled by lithofacies (Sh: Shale, SS: Sandstone, SiS:Siltstone, LS: Limestone).

Figure 6 display cross plots of measured compressional sonic versus conventional logs, categorized by lithofacies. The plot illustrates the correlation between compressional sonic velocities and various conventional log measurements, highlighting distinct trends across different lithofacies types. This analysis helps identify relationships between the sonic properties and geological features, providing valuable insights for petrophysical interpretation and well log analysis. Each data point is color-coded according to its corresponding lithofacies, allowing for a clear visual differentiation of lithological influences on the sonic log characteristics.

Handling categorical features such as litho-facies is an important preprocessing step before proceeding to modeling in machine learning. Litho-facies log have been transformed using one-hot encoding technique which replace each category with binary variable. Each category becomes a separate binary feature, with a value of 1 indicating the presence of that category and 0 otherwise. This method is suitable for litho-facies log.

Data normalization or standardization is a critical preprocessing step which improves the model performance, convergence, interpretability, and robustness^27^. Normalization also, ensures that all features contribute proportionally, facilitating a fair comparison and improving the model’s ability to learn. It is worth to mention that, normalization is mandatory for SVM, KNN and DNN, while it is not mandatory for XGboost and Catboost but it can affect the model results. The features have been normalized using standard scaling, as outlined in the following equation:

2 $$x^{\prime} = \frac{{x - \overline{x}}}{\sigma }$$

where $x^{\prime }$ is the normalized feature, $x$ is the original feature value, $\overline{x}$ is the mean of the feature and $\sigma$ is the standard deviation of the feature.

A classic and popular method for estimating the performance of ML models is holdout cross-validation in which the data is divided into training set and testing set^28^. In addition to the holdout cross-validation evaluation method, a blind well will be used for practical model evaluation. Splitting the data were done by dividing the five wells with complete data into two sets: a training set consisting of four wells and a blind test set consisting of the fifth well. This splitting allows for the training and evaluation of ML models on independent datasets. The training data itself were divided into train set (80% of the training data) and test sets (20% of the training data).

### Machine learning models training

Six distinct machine learning models have been chosen and fine-tuned to forecast the compressional sonic log. These include the ensemble methods (including Random Forest, CatBoost and XGBoost regressors), Support Vector Machines (SVMs), K-nearest neighbors (KNN) and Deep Neural Networks (DNN).

**Fig. 7:**
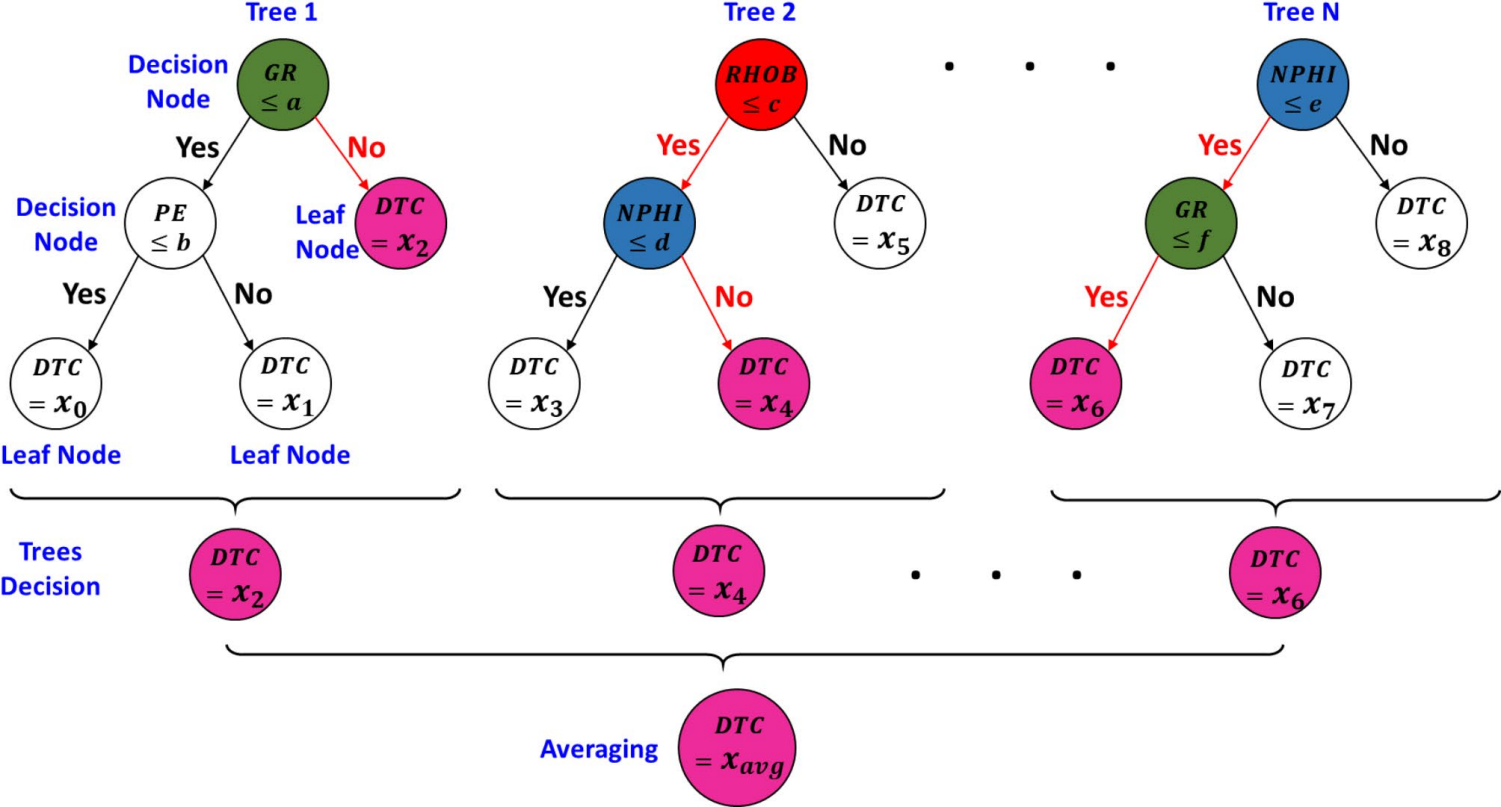
An illustration of the Random Forest regression process for predicting compressional sonic (DTC).

Ensemble methods are considered to be a powerful algorithm in machine learning. This method depends on combining multiple models to produce a stronger regression model than any individual model alone. Random Forest algorithm represents one of these ensemble learning methods and is used for both regression and classification problems^29^. The Random Forest regressor is an ensemble learning technique that extends decision trees by using a method called bagging to improve accuracy and reduce overfitting. It works by creating multiple decision trees, each trained on a randomly selected subset of the training data (with replacement). Additionally, at each split in a tree, a random subset of features is chosen to determine the best split, ensuring diversity among the trees (Fig. 7).

Once all trees are trained, the final prediction is obtained by averaging the outputs of all individual trees, which helps to reduce variance and improve generalization. Since each tree learns from different parts of the data, the model becomes more robust to noise and less prone to overfitting compared to a single decision tree.

CatBoost and XGBoost are both gradient boosting algorithms that build trees sequentially, with each tree correcting the errors of the previous one. Unlike Random Forest (RF), which builds trees independently using bagging. XGBoost is optimized for speed and efficiency, using advanced techniques like tree pruning and parallel computation that relies on traditional gradient boosting for better optimization its scalability and fine-tuning capabilities make it a preferred choice for large-scale datasets^30^. CatBoost is specifically designed to handle categorical features efficiently without needing extensive preprocessing, it also offers a powerful model for handling regression tasks. For regression tasks, CatBoost follows the gradient boosting framework, where each new tree is trained on the residuals of the previous predictions. It uses symmetric decision trees, which ensure balanced splits and improve training efficiency^31^.

K-nearest neighbors (KNN) is a simple and non-parametric supervised learning that it is recommended to be used relatively smaller, well-structured. Instead of fitting a function to the training data, KNN predicts the target value of a new data point by averaging the values of its $k$ nearest neighbors in the feature space (Fig. 7a). Equation 3 is used to calculate the Euclidean distance between two points in an n-dimensional space^32^.

3 $$d\left( {x, x_{i} } \right) = \sqrt {\mathop \sum \limits_{j = 1}^{n} \left( {x_{j} - x_{ij} } \right)^{2} }$$

where $d\left( {x, x_{i} } \right)$ is the Euclidean distance between $x$ (the new data point) and $x_{i}$ (a training sample, $x_{j}$ is the value of feature $j$ in the new data point $x$ and $x_{ij}$ is the value of feature $j$ in the existing training data point $x_{ij}$.

**Fig. 8:**
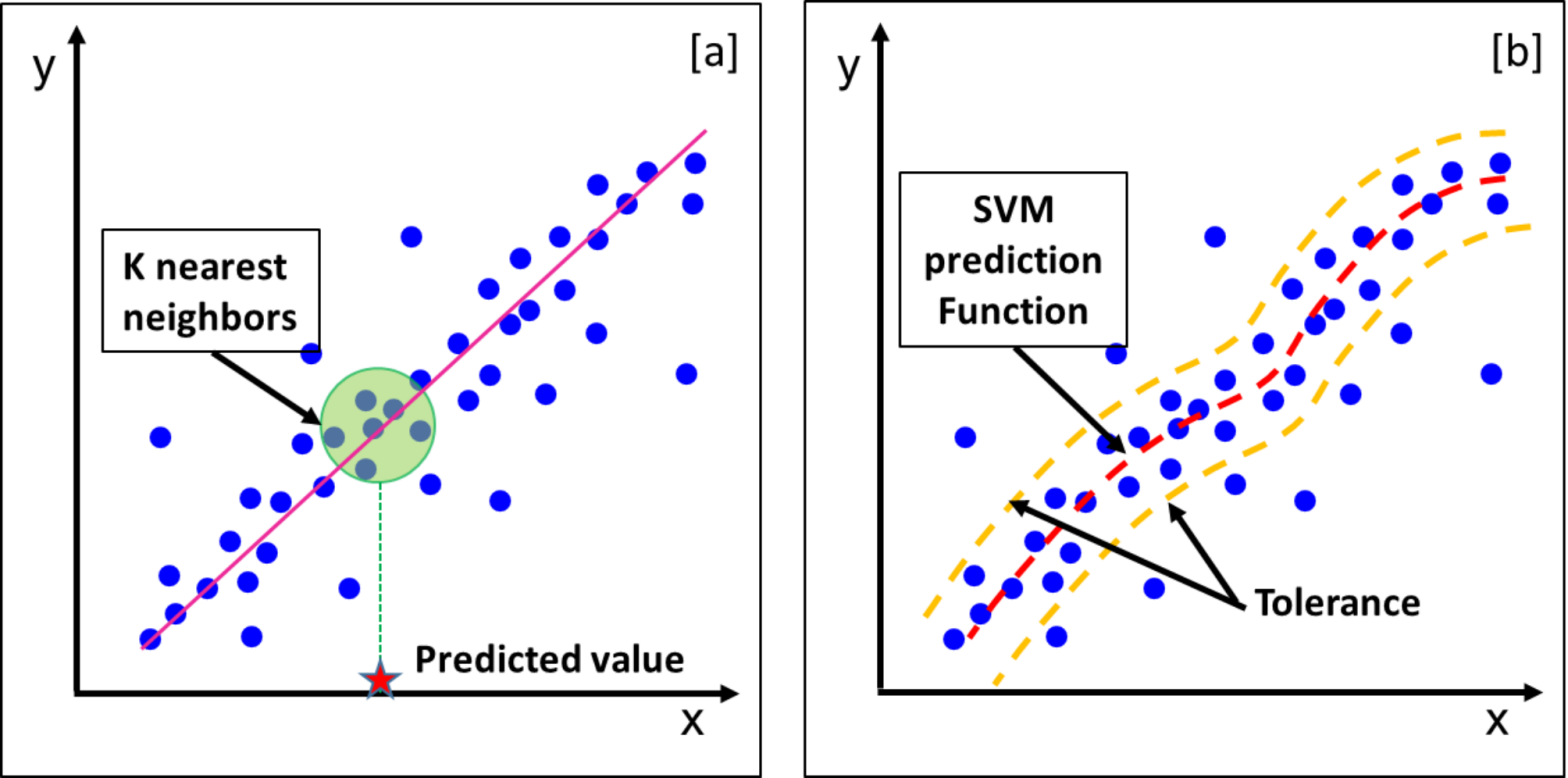
Basic regression concepts (**a**) KNN regression predicts the target value based on the average of its nearest neighbors, represented by the green circle. (**b**) SVM regression fits a function within an acceptable error margin (yellow dashed lines) while minimizing deviations, with the red dashed line representing the regression model.

Support Vector Machines (SVMs) are considered to be a widely used supervised learning algorithm that is suitable for both regression and classification problems. Unlike standard regression techniques that minimize the squared error, SVR aims to find a function that best fits the data while maintaining an acceptable margin of tolerance, denoted by the loss function as shown in Fig. 8b ^33^. SVR employs kernel functions such as linear, polynomial, and radial basis function (RBF) to map input data into a higher-dimensional space, allowing it to capture complex, nonlinear relationships in the data^34^.

**Fig. 9:**
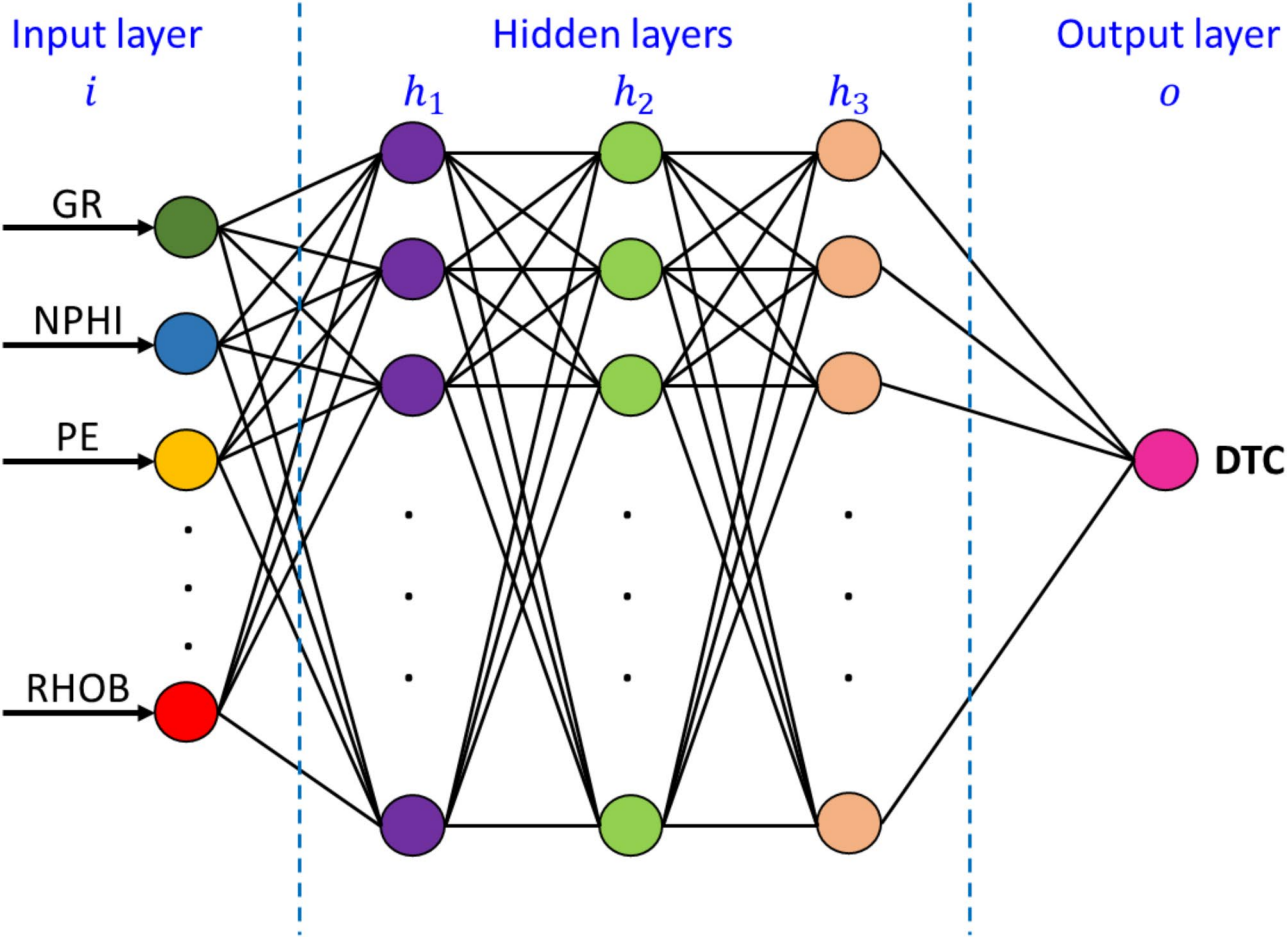
A schematic representation of a deep neural network (DNN) architecture for predicting compressional sonic (DTC).

Deep Neural Networks (DNNs) are known for its strong performance on complex problems due to its ability to uncover the deep relations between the features and the target. A typical DNN architecture consists of an input layer that receives data, multiple hidden layers that progressively transform and refine information, and an output layer that generates predictions (Fig. 9). The depth of these networks enhances their capacity to learn abstract representations, enabling superior generalization and accuracy in predictive modeling^35^.

### Hyperparameters optimization

Hyperparameters are crucial components of machine learning algorithms that influence the behavior and performance of models^36^. Tuning hyperparameters is essential for optimizing model performance, as choosing appropriate values can dramatically enhance the model’s ability to give good result with unseen wells. Hyperparameter tuning is computationally intensive and time-consuming process that requires a careful experimentation and validation to ensure the best possible model performance^37^. Grid search and randomized grid search are both techniques for hyperparameter tuning in machine learning models, but they differ significantly in their approach and advantages. Grid search exhaustively searches through a specified parameter grid, testing all possible combinations of hyperparameters. The main advantage of grid search is its comprehensive nature—by exploring every combination, it ensures that the best parameter configuration is found, as long as the search space is adequately defined.

This exhaustive approach guarantees that no potentially optimal combination is overlooked, making grid search a reliable choice when the parameter space is small or when precise tuning is critical. On the other hand, randomized grid search samples a fixed number of parameter combinations randomly from the search space, making it computationally more efficient, especially for large and complex parameter grids. However, it may miss the optimal combination since it does not explore all possibilities. Randomized grid search can be more effective than grid search in terms of resource utilization when the search space is large because it focuses on exploring a broader range of hyperparameter combinations, potentially achieving similar results with fewer computational resources^36^. The grid search approach for hyperparameter tuning was employed to obtain optimal parameters for all models. Table 3 displays the finest hyperparameters for each method that yielded the most favorable metric outcomes.

**Table 3:** Optimal values of key hyperparameters across all algorithms.

Algorithm	Hyperparameters		
Random forest	Estimators = 430	Min_samples_split = 2	Min_samples_leaf = 1
K-nearest neighbors	Neighbors = 100	p = 5	Weights = ‘distance’
Support vector machines	C = 38	gamma = ‘scale’	kernel = ‘rbf’
CatBoost	Estimators = 1000	Learning rate = 0.1	Max depth = 4
XGBoost	Estimators = 100	Learning rate = 0.1	Max depth = 4
Deep neural network	No of layers = 4	optimizer = ‘Adam’	Learning rate = 0.1

### Model evaluation

The importance of regression model evaluation metrics lies in their ability to quantify the performance of regression models and provide insights into their predictive accuracy. By evaluating a model’s fit and its performance on the blind data, these metrics enable data scientists and researchers to make informed decisions about model selection, parameter tuning, and feature engineering. Regression model evaluation metrics commonly include: mean squared error (MSE) (Eq. 4), root mean squared error (RMSE) (Eq. 5), coefficient of determination (R-squared) (Eq. 6).

4 $$MSE = \frac{{\mathop \sum \nolimits_{i = 1}^{n} \left( {y_{i} - \hat{y}} \right)^{2} }}{n}$$

5 $$RMSE = \sqrt {\frac{{\mathop \sum \nolimits_{i = 1}^{n} \left( {y_{i} - \hat{y}} \right)^{2} }}{n}}$$

6 $$R^{2} = 1 - \frac{{\mathop \sum \nolimits_{i = 1}^{n} \left( {y_{i} - \hat{y}} \right)^{2} }}{{\mathop \sum \nolimits_{i = 1}^{n} \left( {y_{i} - \overline{y}} \right)^{2} }}$$

where $y_{i}$ measured values of DTC is, $\hat{y}$ is predicted values of DTC, $\overline{y}$ is the arithmetic mean, and $n$ is the total number of measured DTC points.

**Fig. 10:**
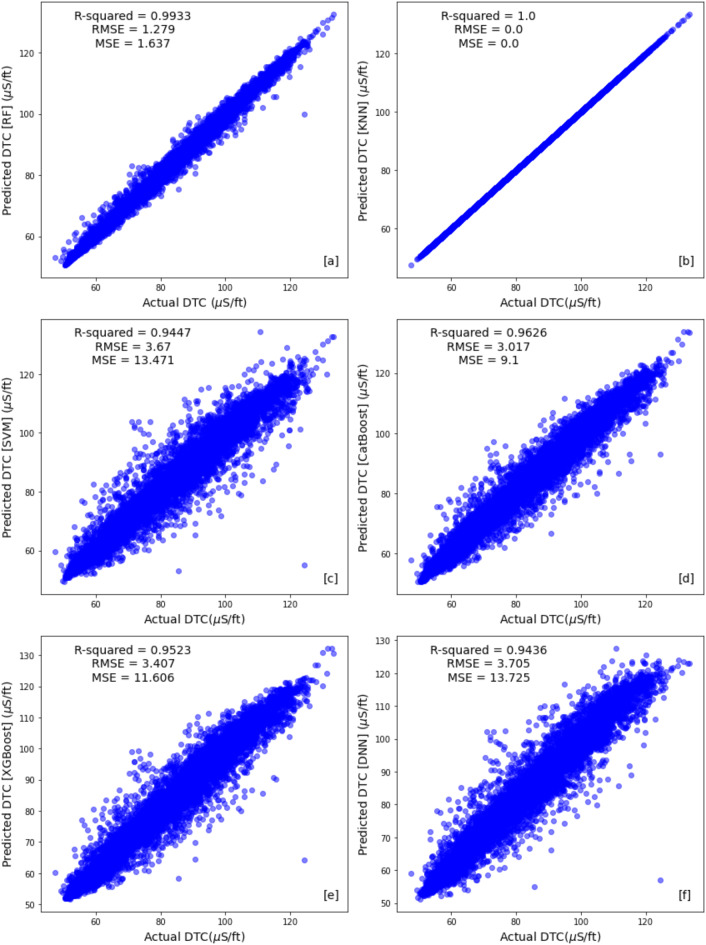
Train set cross plots comparing original compressional sonic logs with synthetic logs generated by (**a**) Random Forest (RF), (**b**) K-nearest Neighbor (KNN), (**c**) Support Vector Machines (SVMs), (**d**) Catboost, (**e**) XGboost and (**f**) Deep Neural Network (DNN) methods.

**Fig. 11:**
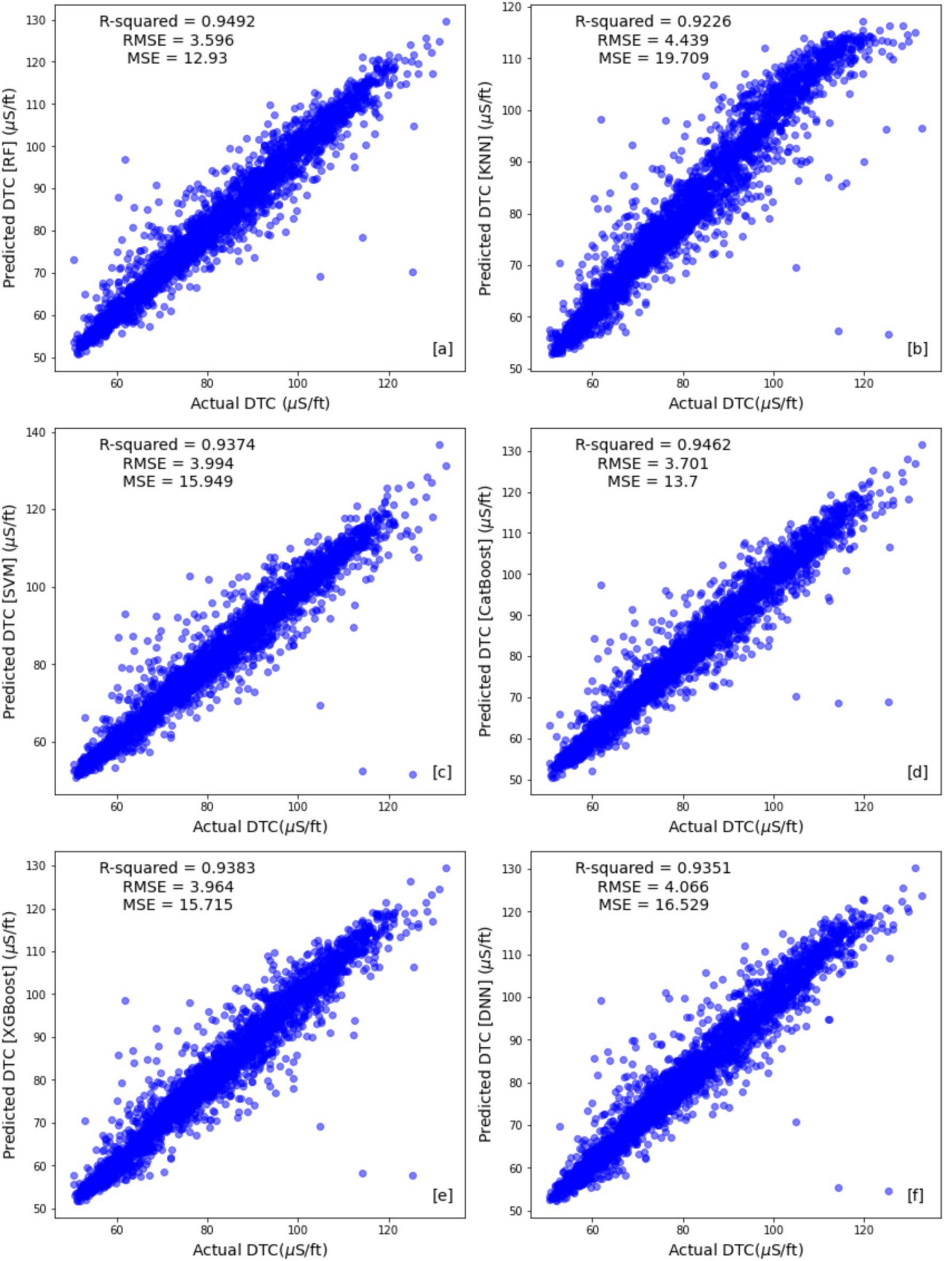
Test set cross plots comparing original compressional sonic logs with synthetic logs generated by (**a**) Random Forest (RF), (**b**) K-nearest Neighbor (KNN), (**c**) Support Vector Machines (SVMs), (**d**) Catboost, (**e**) XGboost and (**f**) Deep Neural Network (DNN) methods.

Table 4 compare the results across different machine learning algorithms using train, test and the blind well test to identify the most promising approach. Figures 10 and 11 illustrate cross plot of the measured compressional sonic log and the predicted sonic log from different machine learning algorithms for training set and testing set respectively. The ensemble methods (Random Forest, CatBoost, and XGBoost) demonstrated superior performance across both the training and test sets. Random Forest achieved the lowest RMSE (1.2794) and the highest R-squared (0.993) on the training set, while its test set RMSE was 3.595 with an R-squared of 0.949 (Figs. 10a and 11a). CatBoost and XGBoost followed closely, with training RMSE values of 3.016 and 3.406 and R-squared scores of 0.962 and 0.952, respectively (Fig. 10d,e). Their test set RMSE values were 3.701 (CatBoost) and 3.964 (XGBoost), with corresponding R-squared scores of 0.938 each, reinforcing their strong predictive capabilities (Fig. 11d,e).

**Table 4:** Performance evaluation metrics for training, testing, and blind well sets across all algorithms.

Algorithm	Set	MSE	RMSE	R-squared
Random forest	Training set	1.636	1.2794	0.993
	Test set	12.930	3.595	0.949
	Blind test well	36.388	6.032	0.8903
K-nearest neighbors	Training set	0.0	0.0	1.0
	Test set	19.636	4.4313	0.922
	Blind test well	49.902	7.064	0.849
Support vector machines	Training set	13.470	3.670	0.944
	Test set	15.939	3.992	0.937
	Blind test well	54.734	7.398	0.8351
CatBoost	Training set	9.099	3.016	0.962
	Test set	13.699	3.701	0.938
	Blind test well	34.219	5.850	0.897
XGBoost	Training set	11.605	3.406	0.952
	Test set	15.715	3.964	0.938
	Blind test well	35.522	5.960	0.893
Deep neural network	Training set	13.725	3.704	0.9436
	Test set	16.392	4.0488	0.935
	Blind test well	49.577	7.041	0.850

In contrast, the non-ensemble methods, including KNN, SVM, and DNN, exhibited comparatively weaker generalization. Notably, KNN showed an RMSE of 0.0 and an R-squared of 1.0 on the training set (Fig. 10b). However, its test set performance dropped significantly, with an RMSE of 4.4313 and an R-squared of 0.922 (Fig. 11b). SVM and DNN also displayed higher training RMSE values of 3.670 and 3.704 with R-squared scores of 0.944 and 0.9436 (Fig. 10c,f), respectively, but struggled in the test set, with RMSE values of 3.992 (SVM) and 4.0488 (DNN) and R-squared scores of 0.937 and 0.935 (Fig. 11c,f). These results highlight the ensemble models’ superior balance between training and testing performance, making them more reliable for predictive modeling.

## Results and discussion

The machine learning models demonstrated promising results in predicting compressional sonic values using conventional logs as input. Feature engineering and data cleaning played pivotal roles in achieving positive outcomes. While ensemble algorithms (random forest, CatBoost, and XGBoost) are theoretically unaffected by scaling and normalization, our findings suggest that it is advisable not to scale or normalize input for these methods, as it may have a detrimental effect on performance. Conversely, Support Vector Machine (SVM), K-Nearest Neighbor (KNN), and Deep Neural Network (DNN) algorithms require input scaling or normalization prior to model training.

**Fig. 12:**
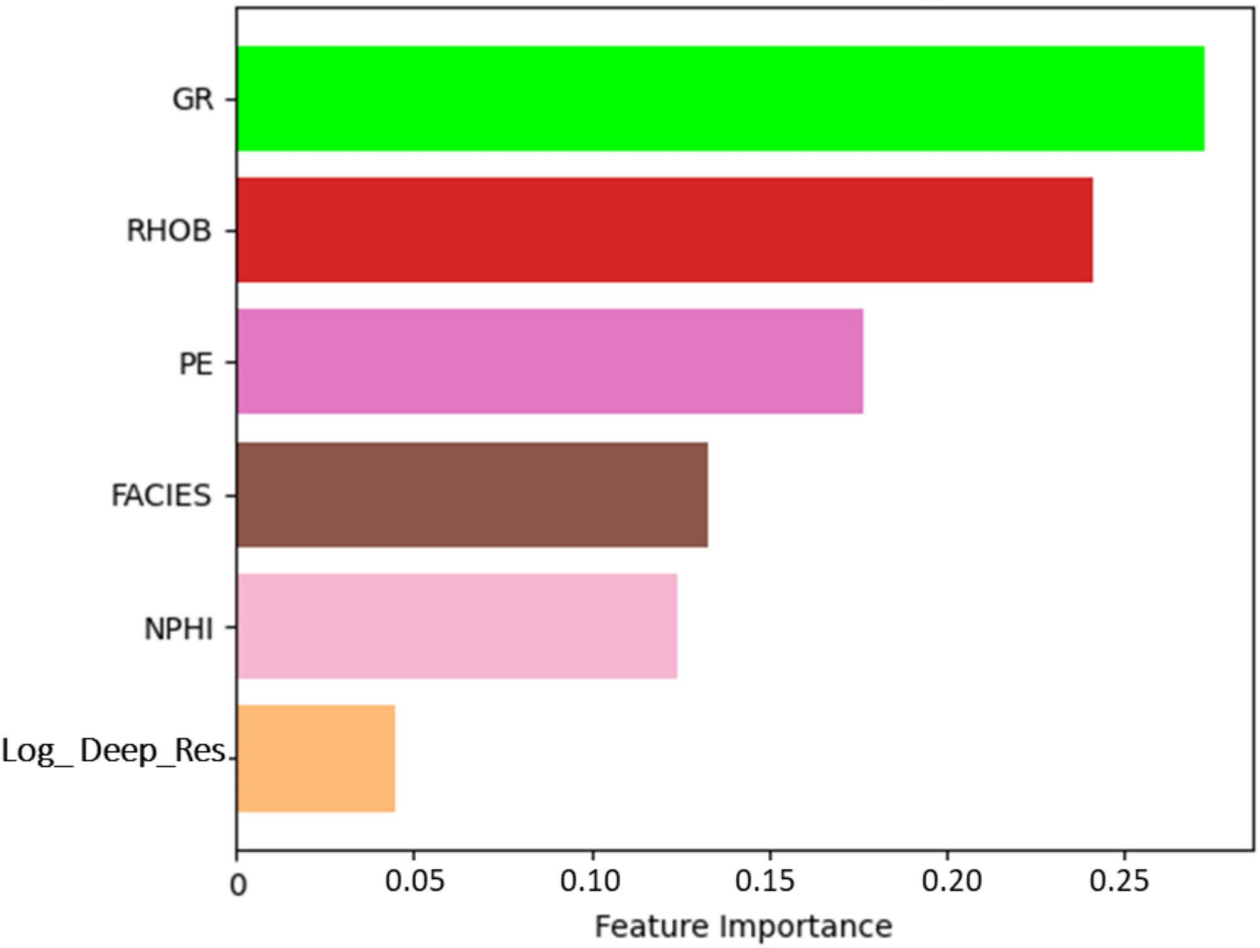
Feature importance analysis for the CatBoost model, highlighting the relative contribution of each input log to the predictive performance.

Feature selection is a main step in machine learning applications for geophysical analysis, as it ensures that the most geologically relevant logs are utilized for accurate prediction of the target. Figure 12 presents the feature importance ranking for the top performing model, CatBoost, illustrating the relative contribution of various geophysical logs to the predictive performance. The Gamma Ray (GR) log exhibits the highest importance, indicating its strong influence on the model’s decision-making process. The Bulk Density (RHOB) log follows closely, suggesting its critical role in distinguishing lithological variations. The Photoelectric Factor (PE) log also holds considerable significance, further reinforcing its utility in characterizing rock properties. Other features, including Facies, Neutron Porosity (NPHI), and logarithm of Deep Resistivity (Log_ Deep_Res), contribute to a lesser extent, yet still provide valuable information for model predictions. The observed ranking highlights the importance of selecting the most relevant logs for robust machine learning models in geophysical applications, ultimately enhancing predictive accuracy and interpretability.

**Fig. 13:**
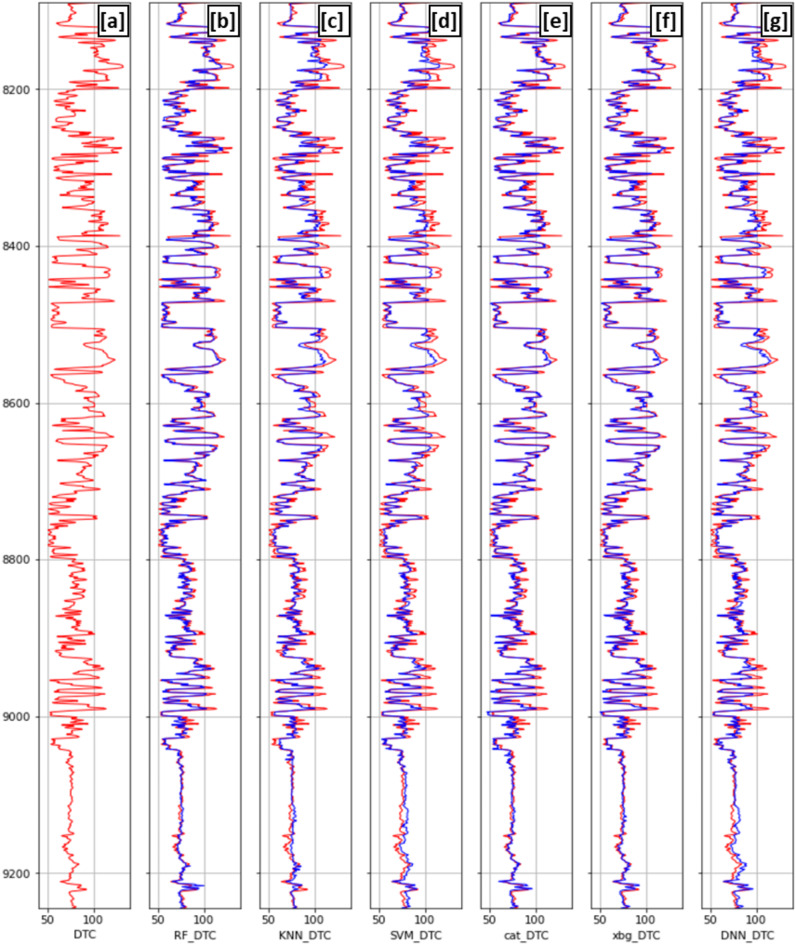
Blind well compressional sonic log for (**a**) the original log of the in red and synthetic logs generated by (**b**) Random Forest, (**c**) K-nearest Neighbor, (**d**) Support Vector Machine, (**e**) Catboost, (**f**) XGBosst and (**g**) Deep Neural Network algorithms in blue versus depth**.**

**Fig. 14:**
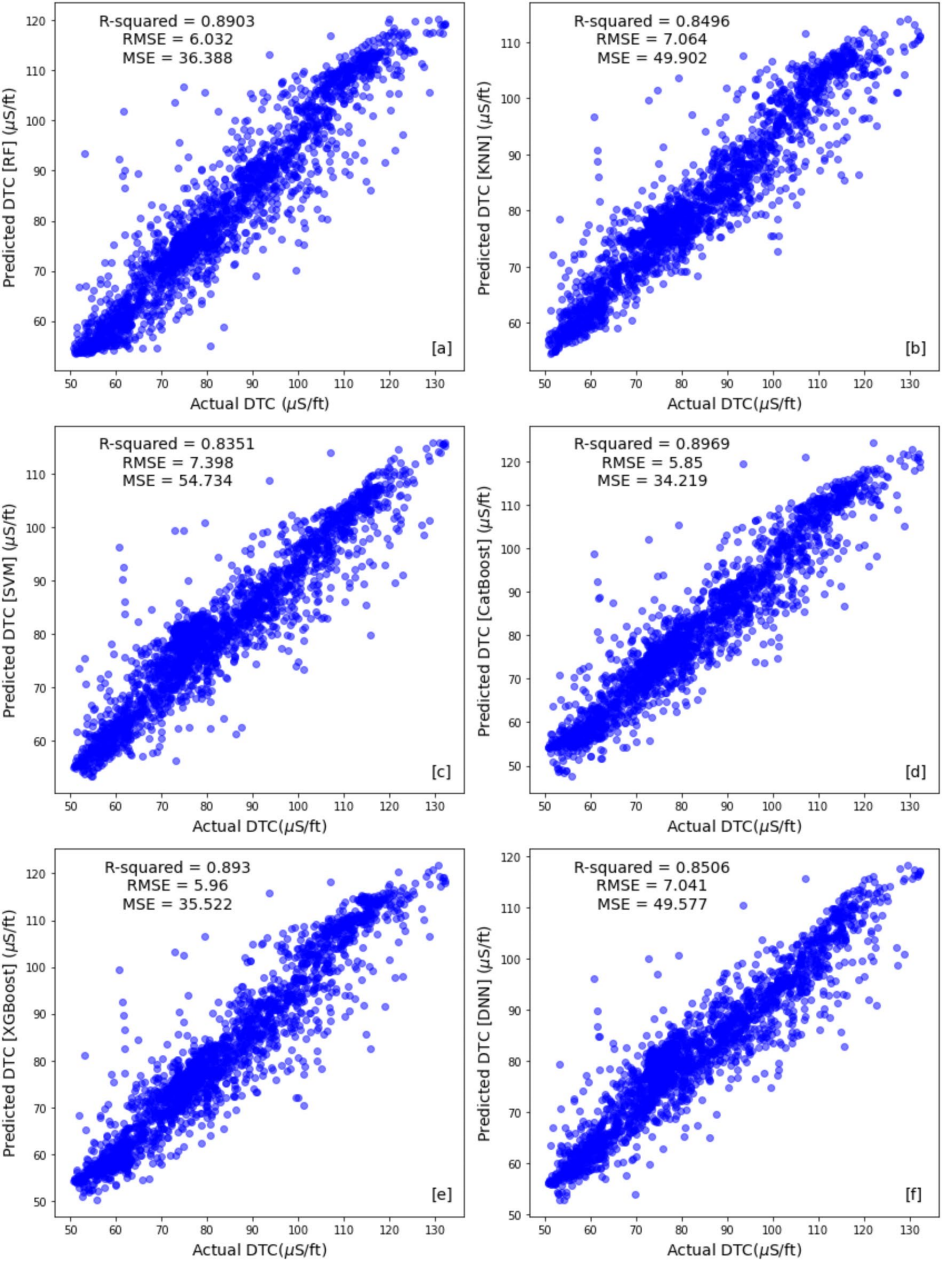
Blind well cross plots comparing original compressional sonic logs with synthetic logs generated by (**a**) Random Forest (RF), (**b**) K-nearest Neighbor (KNN), (**c**) Support Vector Machine (SVMs), (**d**) Catboost, (**e**) XGboost and (**f**) Deep Neural Network (DNN) methods.

Hyperparameter optimization emerged as a critical step, with the potential to significantly enhance model performance. Figure 13 illustrates the performance of different models on the blind test well, comparing their predictions with the original logged compressional sonic log. Overall, all models demonstrate good performance, with ensemble methods (Random Forest, CatBoost, and XGBoost) standing out by providing more accurate predictions in most areas. Blind well testing offers a more comprehensive evaluation beyond the test set, ensuring a robust assessment of model performance. The ensemble algorithms achieved high correlation coefficients (89%–89.6%) and low Root Mean Square Error (5.85–6.03) (Fig. 14a,d,e). In contrast, KNN, SVM, and DNN exhibited relatively lower performance, with RMSE values of 7.064, 7.398, and 7.041, and correlation coefficients of 0.849, 0.8351, and 0.85, respectively (Fig. 14b,c,f). These models showed a tendency to underestimate values compared to the original log. Overall, this study offers a comprehensive workflow for predicting compressional sonic logs, enabling the acquisition of accurate data with minimal error margins.

The CatBoost model exhibited strong predictive performance on both the testing set and the blind test well. Unlike empirical models such as the Wyllie time-average equation and the Raymer-Hunt-Gardner model, which are constrained by specific assumptions, CatBoost offers greater adaptability and accuracy. Furthermore, this approach can be extended to predict other related logs, including shear sonic and elastic property logs, with high reliability.

This workflow facilitates the acquisition of accurate compressional sonic data that can be used for improving subsurface characterization, optimizing reservoir management, and reducing uncertainties in geophysical and petrophysical analyses. Accurate compressional sonic predictions enhance the interpretation of rock properties, fluid distributions, and geomechanical behavior, directly impacting well planning, drilling optimization, and production strategies. By providing a reliable alternative when direct measurements are unavailable or inconsistent, this predictive approach significantly contributes to the efficiency and cost-effectiveness of hydrocarbon exploration and development.

## Conclusion

In conclusion, this study presents a comprehensive workflow for optimizing a machine learning model that is able to predict compressional sonic logs using machine learning models using the available logs. Key findings include the importance of feature engineering, data cleaning, and hyperparameter optimization in achieving accurate predictions. While ensemble algorithms exhibited superior performance, it is essential to take into consideration the specific nature of each model, particularly regarding input scaling and normalization. By integrating blind well testing into the evaluation process, this study enhances the robustness of model performance assessment. The CatBoost model demonstrated superior results, making it a reliable choice for generating accurate compressional sonic logs without incurring additional costs.

## Data Availability

The data supporting the results of this study was obtained from the Egyptian General Petroleum Corporation. Data are however available from authors upon reasonable and with permission of The Egyptian General Petroleum Corporation. Correspondence and requests for materials should be addressed to Khaled Saleh Email: khaledsaleh@gstd.sci.cu.edu.eg.
